# Is immunotherapy in the future of therapeutic management of sarcomas?

**DOI:** 10.1186/s12967-021-02829-y

**Published:** 2021-04-26

**Authors:** Ottavia Clemente, Alessandro Ottaiano, Giuseppe Di Lorenzo, Alessandra Bracigliano, Sabrina Lamia, Lucia Cannella, Antonio Pizzolorusso, Massimiliano Di Marzo, Mariachiara Santorsola, Annarosaria De Chiara, Flavio Fazioli, Salvatore Tafuto

**Affiliations:** 1 Sarcomas and Rare Tumors Unit, Istituto Nazionale Tumori, IRCCS – Fondazione “G. Pascale”, 80131 Naples, Italy; 2 Division of Innovative Therapies, Istituto Nazionale Tumori, IRCCS – Fondazione “G. Pascale”, 80131 Naples, Italy; 3 Nuclear Medicine Unit, Istituto Nazionale Tumori, IRCCS – Fondazione “G. Pascale, 80131 Naples, Italy; 4 Department of Abdominal Oncology, Istituto Nazionale Tumori, IRCCS – Fondazione “G. Pascale”, 80131 Naples, Italy; 5 Histopathology of Lymphomas and Sarcomas SSD, Istituto Nazionale Tumori, IRCCS – Fondazione “G. Pascale”, 80131 Naples, Italy; 6 Orthopedic Oncology Unit, Istituto Nazionale Tumori, IRCCS – Fondazione “G. Pascale”, 80131 Naples, Italy

**Keywords:** Soft tissue sarcoma, Osteosarcoma, Immunotherapy, Anti-cancer vaccine, CAR-T therapy

## Abstract

Sarcomas are rare, ubiquitous and heterogeneous tumors usually treated with surgery, chemotherapy, target therapy, and radiotherapy. However, 25–50% of patients experience local relapses and/or distant metastases after chemotherapy with an overall survival about 12–18 months. Recently, immuno-therapy has revolutionized the cancer treatments with initial indications for non-small cell lung cancer (NSCLC) and melanoma (immune-checkpoint inhibitors).Here, we provide a narrative review on the topic as well as a critical description of the currently available trials on immunotherapy treatments in patients with sarcoma. Given the promising results obtained with anti-PD-1 monoclonal antibodies (pembrolizumab and nivolumab) and CAR-T cells, we strongly believe that these new immunotherapeutic approaches, along with an innovative characterization of tumor genetics, will provide an exciting opportunity to ameliorate the therapeutic management of sarcomas.

## Background

Sarcomas are heterogeneous malignant tumors of mesenchymal origin characterized by more than 50 distinct subtypes. Overall, they are characterized by a low incidence (1% of all malignant tumors in adulthood and 10–15% of all malignant tumors in pediatric age) and in most cases by a poor prognosis. Approximately 15,000 people in the United States are diagnosed with sarcoma every year [1].

Although there are more than 50 types of sarcoma, they can be grouped into two main subtypes: soft tissue sarcomas (STSs) and bone sarcomas (BSs), or osteosarcoma. The term soft tissue refers to tissues that connect, support, or surround other structures and organs of the body. Soft tissue includes muscles, tendons, fibrous tissues, fat, blood vessels, nerves, and synovial tissues (tissues around joints). There are many different types of STSs, however they are grouped together because they share certain microscopic characteristics, produce similar symptoms, and are generally treated in similar ways. Non STSs are osteosarcomas (arising in bone) and chondrosarcoma (arising in cartilage). Ewing’s sarcoma (ES) is a bone sarcomas originating in immature nerve tissue of bone marrow. Osteosarcoma and ES tend to occur more frequently in children and young people, while chondrosarcoma occurs more often in adults [2]. Treatment options and recommendations depend on several factors, including type, stage, and grade of sarcoma, possible side effects, patients’ comorbidities, performance status and preferences.

Surgery is the first-choice treatment for localized tumors to obtain the local control of the disease. In this case, removal of at least 1–3 cm of tissue surrounding the main neoplastic mass (subcutaneous adipose tissue, muscles, bands, bone segments, tracts of vessels arterial or venous) is necessary since sarcoma often produces microscopic satellite nodules (skip metastases) into the healthy tissue around the tumor [3, 4].

Radiotherapy can be used before surgery to both reduce tumor size or to improve the loco-regional radicality after surgery, in case a wide surgical excision cannot be achieved, i.e. voluminous tumor masses, and/or critical locations and/or in the presence of surrounding vital organs. However, after surgery, radiotherapy is often indicated in aggressive subtype of sarcomas in order to reduce the risk of local recurrence.

Chemotherapy is the mainstay treatment in metastatic disease. In localized disease, however, it can be used in the pre-operative phase to reduce the size of the primary tumor as neo-adjuvant therapy or in the post-operative phase in the presence of very aggressive forms, to reduce the risk of both local and distant recurrence (adjuvant therapy). A complete dissertation of chemotherapy is beyond the scope of this review, however, the most active drugs include: anthracyclines and ifosfamide, alone or in combination, decarbonize, gemcitabine, taxanes, etoposide, vinorelbine and trabectedin [5–8].

In recent years, research has prompted a greater understanding of sarcomas subtypes biology making possible to direct the choice of chemotherapy treatment in a “targeted” way. However, despite these improvements, about 25–50% of patients develop recurrent and/or metastatic disease [6, 7] after surgical removal of a primary mass. Complete responses to chemotherapy for metastatic sarcoma are rare and the prognosis is dismal with median survivals from 10 to 15 months [9, 10]. For this reason, the search and the development of new and effective therapies to treat patients with sarcoma is needed.

In the last years, giving (i) the growing evidence that the immune system plays an important role in the control and progression of tumors and (ii) the encouraging results obtained with immunotherapy in some types of tumors, such as non-small cell lung cancer (NSCLC) [11–14] and melanoma [15], it was thought to extend immunotherapy also to sarcomas [2, 16–19].

In this review we will focus on the main immunological therapies for sarcoma, analyzing the clinical research so far conducted. A PubMed and clinicaltrials.gov search with the keywords “sarcoma” and “immunotherapy” was conducted by filtering with “clinical trials”. Like other types of tumors, where research has made significant advances in the immune-therapeutic field, also for sarcomas the possible applications of immunological therapies include: (i) immunologic checkpoint blockade with the targeting of the cytotoxic T-lymphocyte associated protein-4 (CTLA-4), and of the programmed cell death protein 1 (PD-1) axis [16–31], and (ii) therapies with adoptive cell transfer [32–56].

In agreement with D’Angelo et al*.* [17], the main immunological strategies can be grouped into these 3 main categories:

1. Immune checkpoint blockade;
2. Adoptive T cell transfer (ACT);
3. Tumor vaccinations.

## Immune checkpoint inhibitors (ICIs)

Recently, immune checkpoint inhibitors (ICIs), have acquired increasing importance in oncology. These anticancer treatments rekindle the immune response against cancer cells, blocking the interactions between PD-1 (Programmed cell Death-1) and PD-L1 (Programmed cell Death-1 Ligand), a fundamental inhibitory checkpoint that contributes to maintain immune tolerance.

The PD-1 receptor is expressed on the surface of activated T cells. Its ligands, PD-L1 and PD-L2, are expressed on the surface of dendritic cells (DCs) or macrophages, and, in many cases, are also over-expressed on tumor cells. Inhibitory checkpoints ensure that the immune system cells do not mistakenly destroy healthy autologous cells during an immune response (i.e. autoimmune reaction). Cancer cells can exploit these immune checkpoints as a way to evade immune detection and elimination.

By blocking immune checkpoint proteins, including PD-1, PD-L1 and CTLA-4, with monoclonal antibodies, the immune system can overcome cancer’s ability to resist the immune responses and stimulate immune defenses against cancer [14].

Encouraging results have been obtained with ICIs in several types of tumors. In fact, the PD-1- monoclonal antibody pembrolizumab, is indicated for the treatment of non-small cell lung cancer, classical Hodgkin lymphoma, primary mediastinal large B-cell lymphoma, urothelial carcinoma [11–15]. As a consequence, efficacy of pembrolizumab has been tested in sarcomas [16–19]; however, only few patients respond to immunotherapy. Therefore, it is warranted to understand how to identify the potential responders through the evaluation of specific biomarkers, including PD-1/PD-L1expression, TMB (Tumor Mutation Burden), MSI (MicroSatellite Instability). In sarcomas, the identification of predictive biomarkers is challenging and complex because of their extreme heterogeneity. In fact, the data available so far are limited, and in some cases even controversial and downsized [20–28]. For example, D’Angelo et al*.* have found in a series of liposarcoma patients, a high rate of PD-L1 expression (41%, 7/17) [21], while Van Der Graaf et al*.* indicate only 1.6% of expression (1/64) [23].

Furthermore, PD-L1 expression was 35.5% (22/64) in osteosarcomas [14], 75% in synovial sarcomas, 75% in dedifferentiated chondrosarcomas, while 0% in well differentiated chondrosarcomas [20, 21]. However, as emphasized by Liang et al*.*, it remains to be clarified if the PD-L1 expression predicts treatment outcomes in sarcomas, as there are conflicting data on this issue [25]. Thus, the role of PD-L1 expression in sarcomas remains to be elucidated.

In the clinical trials available with ICIs, in particular pembrolizumab or nivolumab, some partial responses were reported, but the number of patients enrolled is too low for being statistically significant. Thus, antitumor activity and efficacy must be further evaluated in larger cohorts.

### Monotherapy

The most important clinical trial regarding the immunotherapy in sarcoma patients is “SARC028", an open-label, single arm, phase 2 study, in which 86 patients with STSs or BSs (40 for each arm) from 12 academic centers in the USA were treated with pembrolizumab at 200 mg intravenously every 3 weeks. This trial, showed that the ORR (objective response rate) was 18% and the 12-week Progression-Free Survival (PFS) 55% (95% CI 42–71%) with a median follow-up of 14.5 months. The best response was seen among patients with undifferentiated pleomorphic sarcoma (UPS), with an ORR of 40%, which included 1 complete response (CR) and 3 partial responses (PRs) among 10 patients. There were 2 and 1 PRs among patients with dedifferentiated liposarcoma (DDLPS) and synovial sarcoma (SS), respectively, and no CRs. There were no responses among patients with leiomyosarcoma (LMS). In BSs, the ORR was 5% and the 12-week PFS 28% (95% CI 14–41%) at a median follow-up of 12.3 months. There was 1 PR each among patients with osteosarcoma and CS, and no responses among patients with ES. There were no CRs in the BS cohort. These results suggest that STSs are more responsive to pembrolizumab than BSs [18]. Among pre-pembrolizumab biopsies, a 4% of tumors was found positive for PD-L1 expression, infiltrated by CD8+ T-cells, and had UPS histotype.

A phase II study of anti-CTLA4 antibody in advanced synovial sarcoma patients (NCT00140855), has produced unsuccessful results and it was terminated and discontinued due to poor accrual. All patients were treated with ipilimumab, a monoclonal antibody that blocks cytotoxic T-lymphocyte antigen 4 (CTLA-4) and received FDA approval for patients with previously treated advanced melanoma in 2011. In this clinical trial, the patients were treated every 3 weeks for three cycles and then re-treated [57]. Patients’ blood was collected to detect the expression levels of the NY-ESO-1 (New York esophageal squamous cell carcinoma 1) protein given its putative role in inducing humoral and cellular immune responses. NY-ESO-1 is expressed in germ and placental cells, and it presents no/low expression in adult normal tissues. Interestingly, it is over-expressed in many tumors, such as melanoma, ovarian, lung, and bladder cancer [58, 59] and in some types of sarcoma, in particular in synovial sarcomas [60] and in liposarcomas. The study, however, was early discontinued because all patients experienced disease progression after 3 cycles of therapy and no substantial differences of NY-ESO-1 expression were observed before and after treatment.

### Combinations

A retrospective analysis of Paoluzzi et al*.* [19], was conducted to evaluate the anti-tumor activity of nivolumab, an anti PD-1 antibody, on 28 patients, with metastatic or unresectable STSs (24) or BSs (4). All patients had received a prior treatment with pazopanib, a tyrosine kinase inhibitor. At disease progression some patients were treated with nivolumab alone, while others with the combination of pazopanib and nivolumab. These authors reported 3 partial responses and 9 disease stabilization. Among the responsive patients, the first was affected by a dedifferentiated chondrosarcoma (DC), received only nivolumab and his tumor had a PD-L1 expression of 20%. The second was affected by an osteosarcoma of left maxilla, and he was treated with a combination of nivolumab and pazopanib. The patient reported a minimal clinical response to nivolumab alone, and pazopanib was then added. After 1 month of pazopanib, her facial lesion significantly regressed allowing a surgical resection. At the time of resection, the tumor showed extensive necrosis with negative margins. PD-L1 expression in this patient was < 5%. The third responding patient was affected by an epithelioid sarcoma (EpS) metastatic to the lung and progressing on pazopanib, he was treated with both pazopanib and nivolumab. This patient had a PR after four cycles of nivolumab; PD occurred with a new lesion in the left lung after four additional cycles. He had further PD in the lung after four more cycles of nivolumab that was finally stopped. This data seems promising for the nivolumab treatment alone or in combination with the tyrosine kinase inhibitor but it needs to be confirmed prospectively on a larger cohort.

A very interesting retrospective study conducted on patients with metastatic STSs using ICIs was conducted by Monga et al*.* [61]. Eighty-eight patients from 4 USA institutions with STSs, treated with a median of two previous therapies, received pembrolizumab (47 patients), nivolumab (6), ipilimumab (1), combination therapy ipilimumab and nivolumab (27). Results were CR in a patient with UPS, PR in 20 patients (7 UPS, 9 leiomyosarcoma), SD in 28 patients. Median progression-free survival (PFS) was 4.1 months, median overall survival 19.1 months.

Patients treated with pembrolizumab monotherapy had an overall survival of 19.1 months and one patient achieved CR. The group of patients treated with a combination therapy nivolumab/ipilimumab showed an overall response of 37% of patients, treatment with nivolumab monotherapy did not yield antitumor responses.

From this retrospective study, it emerges that anti-PD-1 therapy in metastatic STSs induces an antitumor response in some sarcoma subtypes, such as UPS and LMS. The response is observed with the combination of ipilimumab/nivolumab or with pembrolizumab monotherapy.

During the 2020 ASCO meeting, interesting preliminary results of two clinical trials in this context were presented. The first was a phase 2, randomized study (Alliance A091401-ClinicalTrials.gov Identifier: NCT02500797), open-label, multicenter study, to understand if nivolumab worked better with or without ipilimumab in treating patients with metastatic or unresectable sarcoma [62]. The study showed a confirmed response rate of 5% in patients treated with monotherapy nivolumab and 16% in those treated with the combination of nivolumab plus ipilimumab [63]. Tumor responses were observed in patients with UPS, myxofibrosarcoma, leiomyosarcoma, and alveolar soft part sarcoma (ASPS). Efficacy results were also shown in 3 expansion cohorts of gastrointestinal stromal tumor (GIST), UPS, and de-differentiated liposarcoma (DDLPS). The primary end point of the study, 6-month response rate, was reached in the DDLPS and UPS, in patients treated with the combination of nivolumab plus ipilimumab, but not in patients treated with nivolumab alone. In 79 patients of the expansion cohorts, results have shown an objective response rate (ORR) of 28.6% and 14.3% in UPS and DDLPS treated with nivolumab plus ipilimumab versus 7.7% and 7.6% with nivolumab alone, respectively. Also, in terms of median PFS and median OS, the combined treatment nivolumab plus ipilimumab gave better results than nivolumab alone. The PFS in patients treated with the combination of two drugs was 2.9 (in GISTs), 5.5 (in DDLS), 2.7 months (in UPS) versus 1.5–4.6–1.5 months in patients treated with nivolumab alone. The median OS was 2.7(in GISTs) and 13.1 (in DDLS) in patients treated with the drug combination versus 9.1 and 8.1, respectively, in those treated with nivolumab alone.

The second is a phase II, randomized, non-comparative trial, to evaluate nivolumab or nivolumab plus ipilimumab with or without radiation therapy in patients with surgically resectable UPS and DDLPS (NCT03307616) [64]. Secondary end-points of the trial included objective response rate (ORR), 12- and 24-month recurrence-free survival, safety, and patient-reported outcomes. Twenty-four patients were included in the study. Fourteen with surgically resectable DDLPS were treated with nivolumab (cohort A), or ipilimumab plus nivolumab in combination (cohort B); 9 patients with surgically resectable UPS received nivolumab for 1 cycle followed by 50 Gy of radiation therapy plus nivolumab once for other 3 cycles (cohort C) or ipilimumab plus nivolumab for 1 cycle followed by 50 Gy of radiation therapy plus nivolumab once every 2 weeks for a total of 6 cycles (cohort D).

This trial is extremely interesting, because in addition to evaluate the efficacy of nivolumab alone or in combination with ipilimumab, it aims to evaluate the impact of radiation on hyalinization pattern in some sarcoma subtypes. A significant clinical activity in patients with UPS who received nivolumab plus radiotherapy and nivolumab in combination with ipilimumab plus radiotherapy was documented, with a median hyalinization rate of 93%; these data compare favorably with the 5% to 10% median hyalinization rate seen with historical controls. However, limited responses were observed in the DDLPS cohorts, with a median hyalinization rate of 8.75%. In conclusion, the addition of radiotherapy to nivolumab alone or to nivolumab plus ipilimumab treatment has significant clinical activity in UPS. Larger studies to evaluate nivolumab plus radiation treatment are warranted (Table 1).

**Table 1:** Ongoing clinical trials with ICIs in sarcoma

NCI trial number	Drug	Type of sarcoma	Phase	Status
NCT02406781	Pembrolizumab + Metronomic Cyclophosphamide	Advanced sarcomas (Osteosarcoma Leiomyosarcoma + Undifferentiated + soft tissue sarcoma)	II	Recruiting
NCT03123276	Pembrolizumab + gemcitabine	Leiomyosarcoma and Undifferentiated Pleomorphic sarcoma	I/II	Recruiting
NCT03338959	Pembrolizumab + Radiation Therapy	Soft tissue sarcoma	I/II	Recruiting
NCT03092323	Pembrolizumab with radiotherapy, followed by surgical resection versus radiotherapy	Soft tissue sarcoma	II	Recruiting
NCT03069378	Pembrolizumab + talimogene laherparepvec (T-VEC)	Advanced sarcomas	II	Recruiting
NCT03056001	Pembrolizumab + doxorubicin	Soft tissue sarcoma	II	Active, not recruiting
NCT03126591	Pembrolizumab + Olaratumab	Soft tissue sarcoma	I	Active, not recruiting
NCT02636725	Pembrolizumab + axitinib	Alveolar soft + soft tissue sarcoma	II	Active, not recruiting
NCT03414229	Pembrolizumab + Epacadostat	Sarcoma	II	Active, not recruiting
NCT03899805	Pembrolizumab + Eribulin	Liposarcoma Leiomyosarcoma Undifferentiated Pleomorphic sarcoma	II	Recruiting
NCT03469804	Pembrolizumab	Kaposi sarcoma	II	Recruiting
NCT02888665	pembrolizumab + doxorubicin	Sarcoma	II	Active, not recruiting
NCT03123276	Pembrolizumab + gemcitabine	Leiomyosarcoma and Undifferentiated Pleomorphic Sarcoma	I/II	Recruiting
NCT03013127	Pembrolizumab	Osteosarcoma	II	Recruiting
NCT03219671	Nivolumab and Ipilimumab	Classic Kaposi sarcoma	II	Recruiting
NCT03886311	Nivolumab + Talimogene laherparepvec + Trabectedin	Sarcoma	II	Recruiting
NCT03282344	Nivolumab + NKTR-214	Metastatic and/or locally advanced osteosarcoma	II	Active, not recruiting
NCT04118166	Nivolumab + Ipilimumab + Cryotherapy	Soft tissue sarcoma	II	Recruiting
NCT04095208	Nivolumab + Relatlimab	Soft tissue sarcoma	II	Recruiting
NCT03590210	Nivolumab + Trabectedin	Metastatic soft tissue sarcoma	II	Recruiting
NCT04535713	Nivolumab, Gemcitabine, Doxorubicin, Docetaxel	Advanced sarcoma	II	Not recruiting yet (Sep 2020)
NCT03277924	Nivolumab + sunitinib	Advanced soft tissue and bone sarcomas	I/II	Recruiting
NCT03138161	Trabectedin, Ipilimumab and Nivolumab	Soft tissue sarcoma	I/II	Recruiting
NCT03190174	Nivolumab and ABI-009 (mTOR inhibitor)	Advanced sarcoma	I/II	Recruiting
NCT02982486	Nivolumab + Ipilimumab	Sarcoma	II	Not recruiting
NCT04339738	Paclitaxel with and Without Nivolumab in Taxane Naive, and Nivolumab and Cabozantinib in Taxane Pretreated Subjects with Angiosarcoma	Soft tissue sarcoma	II	Recruiting
NCT04165330	Nivolumab + AL3818 (anlotinib)	Metastatic and advanced sarcoma	I/II	Recruiting
NCT03628209	Nivolumab or Nivolumab and Azacitidine	Osteosarcoma	I/II	Recruiting
NCT04149275	Nivolumab + cabozantinib + ipilimumab	Carcinosarcomas (ovary, uterus, vagina)	II	not recruiting yet
NCT02428192	Nivolumab in combination with ipilimumab	Advanced leiomyosarcoma of the uterus	II	Active, not recruiting
NCT03548428	Atezolizumab + radiation	Sarcoma	II	Recruiting
NCT04216953	Atezolizumab + cobimetinib (MEK inhibitor)	Advanced and/or Metastatic soft Tissue sarcoma	I/II	Recruiting
NCT03474094	Atezolizumab + radiotherapy	Soft tissue sarcomas	II	Active, not recruiting

## Adoptive cell therapy (ACT)

ACT is an encouraging and innovative immunological strategy in tumor treatment. The goal of this strategy is either 1. to induce a more targeted and specific immune responses or2. to reactivate the immune system, which is evaded in different tumors.

The three principal ACTs used for cancer immunotherapy [65] are:

1. T cells therapy;
2. CAR-T cells therapy;
3. T cell receptor (TCR) T cells therapy.

### T cells therapy

T-cells-based therapy uses TILs (Tumour Infiltrating Lymphocytes) from the tumor to treat the tumor itself. In particular, TILs are collected, activated and expanded ex vivo, subsequently a huge number of these activated and expanded T cells are re-infused into the patients to induce an effective anti-tumor response. The reinfusion is usually preceded by a lympho-depleting regimen with cyclophosphamide and fludarabine in order to deplete endogenous T-cells and Tregs (T regulatory cells) that may suppress the proliferation of the infused T-cells [32, 33]. The presence of TILs in residual tumor, after neoadjuvant chemotherapy (NACT), is strongly correlated with a better prognosis both in patients with triple-negative breast cancer [34, 35] and in those with advanced metastatic melanoma [33]. The efficacy of TILs therapy has been shown in some different clinical trials. In particular, in a study performed on a population of 21 patients affected by metastatic uveal melanoma encouraging results were obtained. In fact, 7 out of 20 evaluable patients treated with expansion/reinfusion of TILs demonstrated objective tumour regression. Six achieved a partial response, whereas only one a complete response [33].

In sarcomas, the main data on TILs concern their role as predictive and prognostic biomarker [36, 37, 41, 42]. However, an interesting study conducted on a population of 70 sarcoma patients by Mullinax et al*. *demonstrated the feasibility of expanding TILs extracted from STS biopsies in co-cultures with autologous tumors. The main phenotype reported on these samples was CD3 + T lymphocytes. They showed that TILs responded to the autologous tumor when reinfused into the patients [43].

Several clinical trials reported that the TILs presence increases the pathological response and the overall survival, emphasizing the possible role of TILs as potential predictive and prognostic marker in immunological therapies [34, 35]. TILs have been described in various cancers [36–42]: melanoma, carcinoma, breast, ovarian, prostate, head and neck, bladder, esophageal, lung, colorectal cancer and also in some type of sarcoma. In a comparative study, Bach et al*.* reported that TILs are present in about 35% of sarcoma patients [36]. In particular, the subtypes of sarcoma in which the presence of TILs have been observed are GIST, STS, ES, osteosarcoma and uterine sarcomas, even though their effect and potential consideration as predictive markers of response to immunological therapies is not clear at all [37–39].

In GISTs, the highly activated CD3+ TILs have been observed in the tumor area and correlated with improved PFS in multivariate analyses. In the same tumor a considerable density of NK (Natural Killer) CD3- cells were also found, in different areas compared to those containing CD3 + cells, but independently predicting the PFS. Probably CD3+ TILs and NKs contribute to immunosurveillance in GISTs in different ways [40].

For non-GIST STSs, the impact of TILs is variable and difficult to define due to the heterogeneity of sarcoma subtypes. In a study of 249 non-GIST STS patients, Sorbye et al*.* investigated the possible correlation between infiltrating lymphocytes and overall survival. They observed that increased CD20+ lymphocytes in sarcomas with wide resection margins were correlated with better survival [41]. However, controversial data are present in literature. In a study conducted on 50 patients with sarcoma[21], D’Angelo et al*.* evaluated the expression of TILs and PD-L1 on tumor biopsies. They did not observe a significant association between the expression of TILs and PD-L1, the clinical features of the tumor and overall survival. They reported a high percentage of TILs (98%) in tumor samples, mainly CD3+ lymphocytes; although CD8+ and CD4+ lymphocytes were also expressed. They found a higher percentage (41%) of CD3+ TILs in GISTs (9/22) and low density of TILs expression in LMS, synovial sarcoma, chondrosarcoma and liposarcoma. In tumors with higher amounts of infiltrating CD8+ or CD+ , cells were more likely to express PD-L1 and PD-1. Considering the controversial data, the authors suggested that further investigation is necessary, given the small number of samples as well as a greater standardization of detection methods regarding PD-L1. Their results are also in contrast with the data reported in a meta-analysis conducted by Gooden et al*.*, in which the effect of TILs in different tumors is emphasized. In fact, they reported that CD3+ and CD8+ lymphocytes infiltrating the tumor had a positive impact on survival [42].

### CAR-T cells therapy

CAR-T (chimeric antigen receptor T) cells therapy is part of adoptive T cell transfer. It consists on taking patients’ immune cells, modifying, expanding, and reintroducing them into the patients, where they can recognize and eliminate cancer cells.

A complete dissertation on CAR-T structure is beyond the scope of this review. Here, we will focus on their potential role as a promising new therapeutic strategy.

**Fig. 1:**
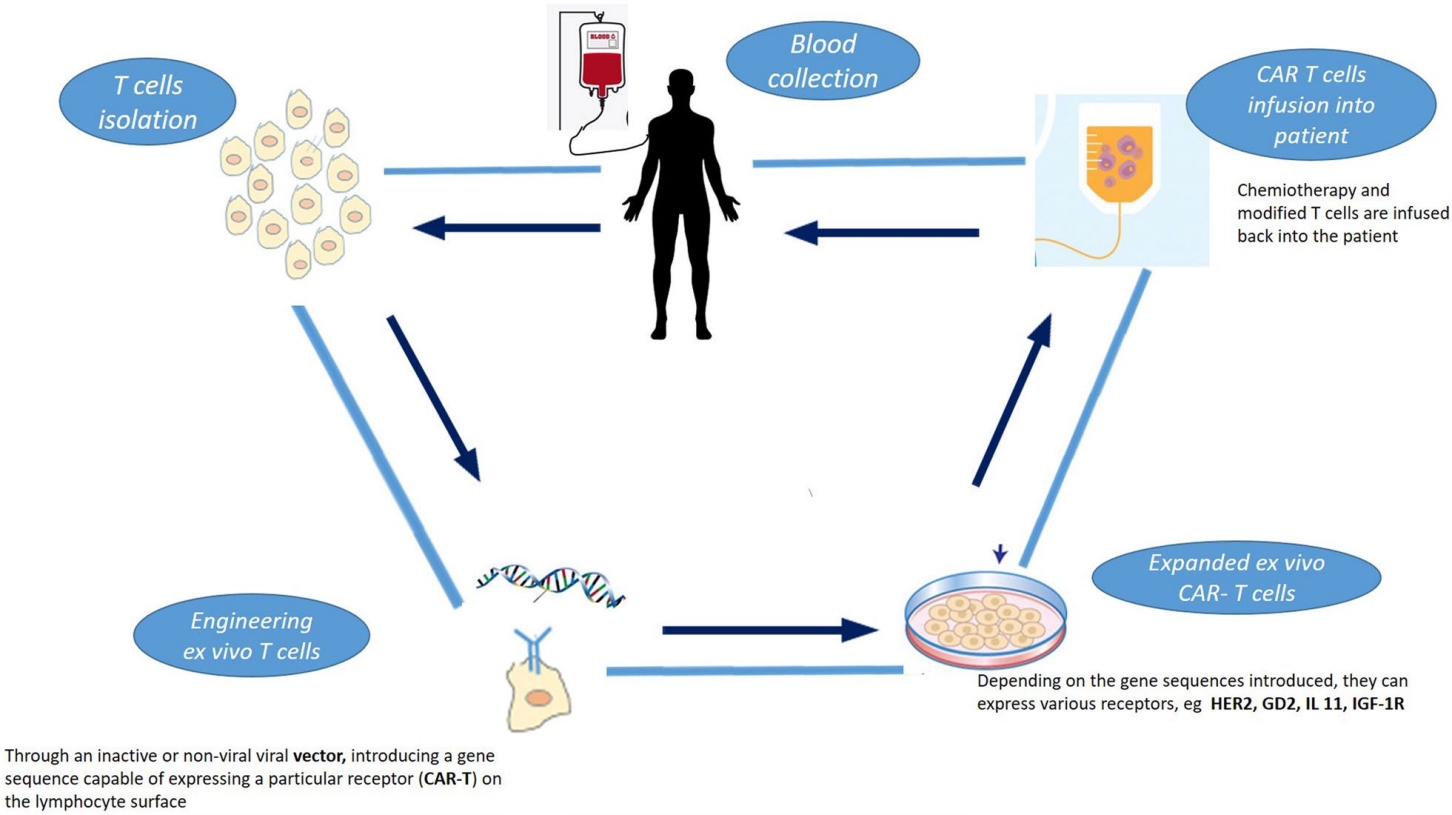
CAR-T cells therapy. Patients’ autologous T cells, can be actively extracted through leukapheresis, reprogrammed ex vivo through an inactive or non-viral vector to introduce a gene sequence capable of expressing a particular receptor (CAR-T) on the lymphocyte surface. Depending on the gene sequences introduced, CAR-T cells can express various receptors binding specific antigen tumor related (HER2, GD2, IL11). CAR-T cells are then selected, "expanded" ex vivo, and reinfused into the host

CAR-T cells therapies are gene therapies aimed to modify the DNA of patient's T lymphocytes, making them able to selectively eliminate cancer cells. The mechanism of action consists in engineering ex vivo the patient's own lymphocytes (autologous) by introducing a gene, which codes for a CAR. The simplest structure of CAR consists of (1) an antigen-recognition domain, usually a single-chain variable fragment (scFv) derived from a monoclonal antibody targeting the selected antigen (i.e. CD19), (2) a hinge [usually derived from CD8 or immunoglobulin 4 (Ig4) molecules] that links the recognition site to the transmembrane domain which bridges the membrane, (3) an intracellular domain that typically contains a CD3ζ chain critical for T-cell receptor (TCR) signaling [66]. These genetically modified T lymphocytes are able to bind the tumor antigen without the need for the major MHC complex (Fig. 1).

The absence of MHC restriction in CAR-T cells therapy offers several advantages. It circumvents immune-evasion if MHC expression is modified while maintaining TCR binding affinity and antigenic intracellular processing. Moreover, in recent years, the search of more specific and selective mechanisms has led to the improvement and development of new "generations" of CAR -T cells that contain a nuclear factor of activated T cell response for the inducible transgenic product as IL-12, IL-18, IL-9 [66]. The last modification makes CAR-T more selective and effective. As previously mentioned, the CAR-T cells can induce the expression of different receptor binding specific antigen tumor related, for example: HER2, GD2, IL-11, IGF-1R.

After the encouraging results obtained in clinical trial in treating CD19+ B-cell lymphoma and acute lymphoblastic leukemia, recently, FDA approved CAR-T as a new treatment for these diseases. As reported by Sermer et al*.* [67], CAR-T cell therapy induced complete responses (CRs) in approximately 40% to 60% of aggressive lymphomas, and 60 to 80% in the other forms [68–70]. However, in addition to promising results, severe adverse events, as “cytokine release syndrome” and severe neurotoxicity, were observed in some patients [69]. These promising results led to an extension of study in sarcomas both at preclinical and clinical level. A phase I trial (NCT02107963), conducted on children and young adults with osteosarcoma and GD2+ solid tumors (excluding neuroblastoma), has been completed, but the results have not been published yet. The primary objective was to determine the safety and the anti-tumor activity of a new 3rd generation anti-GD2-CAR, (anti-GD2.28.z.OX40.ICD9) that has the peculiar feature of being combined with a “suicide” switch caspase dimerization domain (ICD9) inducing CAR-T apoptosis in case of toxicity [65]. In this phase I trial, patients received an escalating dose of autologous anti-GD2-CAR, following cyclophosphamide as lympho-depleting regimen. The study evaluated also the use of AP1903, a dimerizing agent, administered to mediate clearance of the genetically engineered cells and resolve toxicity in case of unacceptable toxicity related to anti-GD2-CAR.This construct is directed against GD2, a disialoganglioside involved in signal transduction, proliferation and tumor cell migration [44]. GD2 has also been considered an attractive target for cancer immunotherapy. It is over-expressed on various tumors including neuroblastoma, melanoma, osteosarcoma, ES, and rhabdomyosarcoma, while it is poorly expressed in normal tissue. Furthermore, some studies showed that the median survival time of patients exhibiting ganglioside GD2 expression was significantly shorter than that of patients without ganglioside GD2 expression [44–46]. Other clinical trials, using the autologous anti-GD2-CAR engineered T cells (NCT03635632- NCT04539366- NCT01953900) in subject with advanced sarcomas and neuroblastoma, are currently ongoing.

A phase I clinical trial using intravenous injection of autologous T cells expressing HER2-specific CAR in patients with advanced HER2-positive osteosarcoma has demonstrated encouraging early findings in both pediatric and adult patients with advanced HER2-positive sarcomas (NCT00902044). The trial is ongoing, but not in a recruiting phase; preliminary results were presented during American Association for Cancer Research (AACR) Annual Meeting in 2019. Patients received one dose of autologous HER2-CD28 T cells, transduced with retro-viral encoding HER2-CD28-CD3ζ, a second-generation of CAR-T cells [47] that express the gene HER2 and contains the domain CD28, which stimulates T cells and makes them last longer in the host. The HER2 antigen is chosen for this CAR-T cell model, because it is well known to play a very important role in breast cancer, promoting the growth of cancer cells. Furthermore, it is over-expressed in a variety of cancers, including sarcomas such as medulloblastoma, synovial, osteosarcoma, ES [47–51]. In osteosarcoma, it appears to be associated with a worse response, as shown by Scotland et al*.*, by increasing the expression of a P-glycoprotein, responsible for multidrug resistance [48]. In this trial, a combination of HER2-specific CAR-T cells and chemotherapy were used. Chemotherapy with fludarabine alone or in combination with cyclophosphamide were administered to obtain lympho-depletion and favor the expansion of T cells clones in the body. The preliminary results of the study seem promising, 3 patients had stable disease, and five progressive disease. One pediatric patient with advanced rhabdomyosarcoma had a complete response for 12 months but relapsed and was retreated again with CAR-T cells resulting in a new complete response that lasted for 17 months. One young patient with osteosarcoma with metastasis to the lungs had complete response for 32 months. The patients experienced limited treatment-related toxicities, eight patients developed grade 1–2 cytokine-release syndrome within 24 h of receiving CAR-T cells but they recovered from toxicity within 5 days from starting supportive care (Table 2).

**Table 2:** CAR-T cell clinical trials in patients with various types of sarcomas

NCI Trial Number	Drug used	Study design	Type of sarcoma	Detailed description	Phase	Status
NCT03356782	Sarcoma-specific CAR-T cells + immune checkpoint antibodies	1 infusion, for 1 × 10^6 ~ 1 × 10^7 cells/kg via IV	Osteoid sarcoma, ES	Peripheral blood mononuclear cells (PBMCs) of patients who have CD133, GD2, Muc1, CD117 or other marker positive sarcoma will be obtained through apheresis, and T cells will be activated and modified to sarcoma-specific CAR-T cells	I/II	Recruiting
NCT04433221	Multiple sarcoma-specific CAR-T cells + sarcoma vaccines	1 infusion, CART 1 × 10^6 ~ 1 × 10^7 cells/kg via IV and vaccines 1–5 × 10^6 irradiated cells via subcutaneous injection	Osteoid sarcoma, ES	Patients eligible must have confirmed surface antigens including GD2, PSMA, Her2, CD276. This study combines multiple CAR T cells with low dose chemotherapy, such as doxorubicin to modulate surface PD-L1 level and enhance immunotherapy effect	I/II	Recruiting
NCT00902044	Autologous HER2-specific T cells + Fludarabine + Cyclophosphamide	1 infusion 1 × 10^8/m^2 autologous T cells after lymphodepleting chemotherapy	Sarcoma	Each patient had received one dose of autologous HER2-CD28 T cells. This trial used a combination of chemotherapy and HER2-specific CAR T cells.The patient with SD or a reduction in the size of the tumor,they can receive additional doses of HER2-specific T cells	I	Active, not recruiting (preliminary results available)
NCT03635632	C7R-GD2.CART cells + Cyclophosphamide + Fludarabine	Patients is assigned a dose of GD2-C7R T cells. They are treated before with cyclophosphamide and fludarabine	Neuroblastoma, osteosarcoma, ES, Rhabdomyosarcoma relapsed uveal melanoma Phyllodes breast tumor	These patients are injected with a GD2.C7R T cells, the retroviral vector used contains a gene that can recognize and kill cancer cells (GD2.CAR) and the new gene called C7R that will help these cells survive longer	I	Recruiting
NCT03618381	EGFR806 CAR T cell (second generation)	2 arms of the study: on Arm A, participants receive EGFR 806CAR(2G) -EGFRt, on Arm B EGFR806CAR(2G)-EGFRt and CD19CAR(2G)-T2A-HER2tG	Soft Tissue Sarcoma (ES, synovial sarcoma), osteosarcoma	Subjects receive a single dose of T cells comprised of two different subtypes of T cells (CD4 and CD8 T cells), that have been genetically modified to express the EGFR 806CAR(2G) -EGFRt—second generation. On ARM B patients receive CAR T cells directed at EGFR and CD19, a marker on the surface of B lymphocytes, that presents cells to T cells	I	recruiting
NCT02107963	Anti-GD2-CAR engineered T cells + Cyclophosphamide + AP1903	It was injected an escalating dose of autologous anti-GD2-CAR (1 × 105/106/107 transduced T cells/kg) following cyclophosphamide-based lymphodepletion	Sarcoma Osteosarcoma Neuroblastoma Melanoma	A 3rd generation anti-GD2-CAR, combined with a suicide switch caspase dimerization domain (ICD9), that induces CAR-T apoptosis in the case of several toxicity, was administred to different doses.The trial involved the use of AP1903, a dimerizing agent, administered to mediate clearance of the genetically engineered cells a in case of an unacceptable toxicity related to anti- GD2-CAR	I	Completed (results not available yet)

### T-cell receptor-based therapy

Adoptive T-cell treatment based on T-Cell Receptor (TCR) modifications is another promising approach to effectively target tumors. This therapy utilizes the engineered T lymphocytes specificity for tumor antigens. In brief, the patients’ autologous T cells are extracted through leukapheresis or from tumor tissue, reprogrammed ex vivo through a lentivirus or retrovirus vector encoding a specific TCR gene, and expanded in order to inject a high number of cells into the patient [52]. TCR is a protein complex found on the surface of T lymphocytes, [52]. It is composed of two different protein chains, alpha (α) and beta (β), non-covalently associated to CD3 complex. TCR recognized fragments of antigen, bound to MHC molecules [66]. The binding of TCR to the MHC-antigen complex, in combination with other co-stimulator signals, leads to the activation of the T lymphocyte. The critical point of TCR T cell therapy is the modulation of MHC recognition. In fact, to improve the affinity of TCR for antigen–MHC complex, which is typically weak in the isolated lymphocytes, TCRs are modified ex vivo through the mutagenesis of one or more specific aminoacids within the complementarity-determining regions (CDRs) [53, 54].

An important step in this therapeutic strategy is the identification of tumour-specific antigens. These proteins are absent or have limited expression in normal tissues such as cancer-testis antigens (CTAs). In fact, these antigens are over-expressed by several neoplasms but are not expressed in normal tissues, except at limited level in adult testis (and in the developing fetus); this makes CTAs interesting targets for immunotherapy (as beyond explained in this review in the “Vaccines” section).

In an interesting pilot, phase 1–2, open-label, non-randomized study, a complex and innovative technology, directed towards NY-ESO-1 (in particular NY-ESO-1-1c259) (NCT01343043) was used [55]. In this study, 42 patients with advanced synovial sarcoma were injected with genetically modified autologous T cells expressing NY-ESO1-1c259, an anti-NY-ESO specific HLA-A*02-restricted peptide SLLMWITQC receptor. The study reported that 1 patient achieved complete response, 14 achieved partial response, 24 showed a stable disease (SD), progressive disease was observed in only 3 patients (PD).

In Another Phase I/II study, patients with metastatic melanoma and sarcoma were treated with autologous T lymphocytes. T cells were transduced with TCR gene directed against NY-ESO-1 antigen. The treatment was associated with systemic IL-2 administration, after lympho-depletion. Patients enrolled were supposed to express NY-ESO-1 antigen that is present in about 80% of synovial sarcomas and in 25% of melanomas. For this reason, NY-ESO-1 is considered a good candidate for specific tumor antigen therapies. Four out of 6 patients with synovial sarcoma had a partial response. Thirty-eight percent of sarcoma patients were alive at 5-years follow-up; this represents a good result compared to chemotherapy [56].

Furthermore, the selection of tumour antigens that are absent or little expressed in normal tissues, is extremely important also to reduce immunotoxicities. In fact, TCR T cell therapy can lead to immune reactions against normal tissues and “tumour off” toxicity [52]. These side effects occur because the most known tumour antigens are not exclusively expressed into tumours, thus triggering alloreactivity reactions [71].The mismatch after introduction of TCR chains with endogenous TCRs is the cause of unpredictable TCR toxicities related to the generation of T cells with novel and paradoxical specificities [56].

## Oncolytic virotherapy

Oncolytic virotherapy is a novel and encouraging therapy applied for the treatment of multiple types of cancers [72–74]. Oncolytic virus therapy became a more concrete reality after the progresses of DNA recombinant technologies, which allowed viruses to be safer and more cancer specific, maintaining a low pathogenicity towards normal host cells. This therapy consists of intra-tumoral or intra-venous injection of natural or engineered viruses, where they preferentially infect and kill tumor cells while sparing the normal ones. As the cancer cells are destroyed by oncolysis, new viruses or virions are released along with cytokines (e.g. GM-CSF, IL-2, IFN-gamma, etc.) and tumor antigens that further stimulate the immune system activity against cancer. In particular, tumor antigens released by destroyed cancer cells are processed by APC cells and presented to the CD4+ and CD8+ lymphocytes triggering the immune response that improve tumor destruction [73, 74].

The first oncolytic virus has been approved in 2005 in China. It was a genetically modified H101 adenovirus used for the treatment of head and neck cancer. In Europe, the first oncolytic virus was approved by FDA in October 2015 for melanoma treatment. It is a modified herpes simplex virus known as Talimogene laherparepvec (T-VEC) [75]. To date, several oncolytic viruses have been successfully tested in different types of cancers in phase 2 or 3 clinical trials [76–78], even though their clinical use for the sarcoma treatment is still limited. Below, we report the few experiences available in scientific literature about the experimental and clinical studies performed to identify which viruses can infect sarcomas.

The most oncolytic viruses used in preclinical and clinical studies are T-VEC, HSV1716, NV1020, G207, M032, rRp450. They are used in melanoma, colon, breast, lung and liver tumors and in some sarcomas [79–87]. Particularly, NV1020 and G207 have been used in osteosarcoma but preclinical studies show modest activity [88]. In particular, apre-clinical study on 10 different cell lines of rhabdomyosarcoma, osteosarcoma and ES was performed using both NV1020 and G207 viruses. The studies showed a different activity against the histologic subtypes. In particular, rhabdomyosarcoma and malignant fibrous cells were more sensitive to oncolysis that osteosarcoma cells, which showed an intermediately sensitivity. ES cells were the less susceptible to oncolysis [88].

The efficacy of other viruses was tested on both sarcoma cell cultures and in a mouse model [77]. Among several selected viruses, the rabdovirus MG1 demonstrated the greatest potency in vitro, because it infected about 80% of the test cells. In vivo the MG1 oncolytic treatment led to a significant increase of survival in mice with sarcomas. Furthermore, this study showed that MG1 treatment induced a memory immune response, providing protection against the tumor, suggesting the potential use of rabdovirus MG1 as oncolytic immunotherapy in sarcoma treatment (alone or in combination with other therapeutic strategies).

In a phase I clinical trial, the adenovirus ONYX-015 (dl1520) was administered in patients with advanced sarcoma in combination with standard chemotherapy (mitomycin-C, doxorubicin, and cisplatin) [82].This combination treatment has been tested to increase the efficacy of the engineered adenovirus, in fact, as documented in a previous phase I/II trial in patients with ovarian and colorectal cancer, the administration of the virus alone showed a limited activity [83, 84]. ONYX-015 is a genetically engineered adenovirus in which the E1B-55k and E3B genes are mutated [15]. The E1B protein binds to the tumor suppressor protein p53 [85], leading to its degradation, preventing cell cycle arrest. p53 is known to be mutated in many cancers especially in sarcomas [86]. In a previous pre-clinical study, it was observed that ONYX-015 virus effectively replicates and cause significant cytotoxicity on sarcoma cell lines. From the clinical trial emerged that only one patient out of 5, with a malignant peripheral nerve sheath tumor, had a partial response. However, ONYX-015 has been shown to replicate in sarcoma tissues and can be safely administered in combination with standard chemotherapy in sarcomas. In another preclinical study performed in osteosarcoma, the adenovirus Δ24-RGD showed a strong synergistic anti-tumor effect with cisplatin both in vitro and in vivo [87]. In particular, in orthotopic osteosarcoma animal models, extensive area of necrosis were documented with a safe toxicity profile.

## Cancer vaccines

Cancer vaccines are one of the immunotherapeutic strategies applied to recognize and eliminate cancer cells. Sources of antigens for vaccines can consist on: 1. killed tumor cells, 2. antigens purified from patients, 3. antigens produced in laboratory [58, 89–127].

Despite the increasing attention of researchers and the several clinical trials carried out so far with cancer vaccines in different tumors, limited results have been obtained and up to now only a few vaccines have been approved by FDA. In 2010, the Sipuleucel-T vaccine against hormone-resistant prostate cancer (i.e. no longer sensitive to anti-androgen hormone therapy) was approved by FDA [92]. It is composed of DCs from the patient stimulated with an antigen present in most prostate cancer (PAP- Prostatic Acid Phosphatase) cells. However, Sipuleucel-T was subsequently withdrawn from the market in Europe because the system to produce it was too complex and expensive and its effectiveness was neither confirmed nor satisfactory. Another vaccine approved is Bacillus Calmette-Guérin (BCG), a tuberculosis vaccine that acts as a broad immune stimulant [93]. In 1990, BCG became the first immunotherapy to be approved by FDA and it is still used for the treatment of early-stage bladder cancer. In 2015, a therapeutic anti-cancer vaccine for metastatic unresectable melanoma (T-VEC) was authorized in Europe. As above discussed, it was a weakened and modified form of the herpes simplex virus type 1 (HSV-1) that reproduces in cancer cells determining the release of an immune-stimulating substance (GM-CSF). Thus, stimulated T lymphocytes kill infected cells, breaking them down and transforming the tumor itself in an autologous vaccine [94].

Tumor vaccines for sarcomas so far tested in clinical trials are: tumor specific antigens, specific fusion proteins (e.g. derived from gene translocations), autologous cells (e.g. dendritic cells of the patient), ganglioside (GD).

One of the main critical points in developing a vaccine is the identification of tumor specific antigens. Over-expressed antigens or specific mutated proteins on cell surface are ideal candidates for an anti-cancer vaccine. Some subtypes of sarcomas, despite their strong heterogeneity, can be targets for this therapeutic strategy, because they have specific genetic abnormalities including chromosomal translocations [i.e. the synovial sarcoma, that is characterized by chromosomal translocation (X,18; p11q11)] [90, 91, 95, 96]. Furthermore, sarcomas present other specific antigens, as CTAs, expressed in germline cells; they reduce or disappear in normal cells and are regained and over-expressed in cancer cells (Table 3 shows some vaccine trials in sarcomas).

**Table 3:** Principal vaccines trials in sarcoma treatment

NCI trial number	Drug	Type of sarcoma	Study design	Phase	Status
NCT01803152	Dendritic Cells Vaccine	Metastatic sarcomas	The patient's dendritic cells used for the vaccine are loaded with tumor lysate. There are three intended dose levels of DC cells per treatment. The vaccine is somministred with topical imiquimod (an immune response modifier), with or without the inhibition of MDSC by gemcitabine pre-treatment	I	Active, not recruiting
NCT01241162	Dendritic cell vaccine with adjuvant	Ewings sarcoma, Osteogenic sarcoma, Rhabdomyo sarcoma, Synovial Sarcoma, Neuroblastoma	The dendritic cells used for the vaccine production, was pulsed with overlapping peptides mixes derived from full-length NY-ESO-1, MAGE-A1, and MAGE-A3. The administration of DC vaccine is preceded by decitabine as a demethylating chemotherapy	I	Completed
NCT00027911	NY-ESO-1 peptide vaccine + Sargramostim	Soft tissue sarcoma	Patients receive NY-ESO-1 peptide vaccine and receive sargramostim (GM-CS), recombinant granulocyte macrophage colony-stimulating factor, before every vaccination	I	Terminated
NCT04433221	Multiple CAR-T cells and sarcoma vaccines	Stage III, IV sarcoma patients or recurrent sarcoma patients;	This trial combines CAR T cells with low dose chemotherapy and followed by maintenance sarcoma vaccines. Chemotherapy, as doxorubicin, is used for the purpose of modulating surface PD-L1 level and enhance immunotherapy effects	I/II	Recruiting
NCT01258868	Tumor cell vaccine + ISCOMATRIX Adjuvant	Thoracic sarcomas,mesolthelioma, Esophageal cancer, lung cancer	Patients are vaccinated with autologous tumor cells exposed ex vivo to decitabine,a hypomethylating agent and radiation. Vaccine is administered in conjunction with ISCOMATRIX adjuvant, that induces both humoral and cellular immune responses and with oral celecoxib, an anti-inflammatory	I	Terminated
NCT00020267	MAGE-12 peptide vaccine	Soft tissue sarcoma, ovarian sarcoma, melanoma, Colon, lung, breast cancer	Patients received MAGE-12 peptide vaccine emulsified in Montanide ISA-51 adjuvant subcutaneously (SC) weekly for 4 doses (arm A) or once every 3 weeks for 4 doses (arm B). Patients with PD could receive IL-2, with the purpose of increasing the immune response	I	Completed
NCT04166006	Dendritic cell vaccine + Interleukin-2	Soft tissue sarcoma, Neuroendocrine tumors, rare cancer	Patients inject with autologous DC vaccine, that it is followed by subcutaneous injection daily of IL-2, as adjuvant to increase the efficacy of vaccine	II	Recruiting
NCT01141491	Trivalent ganglioside vaccine + OPT-821	Sarcoma	Patients received the vaccine combined with OPT-821, an immune system stimulant (arm A) or the immune system stimulant alone (arm B). The trivalent vaccine should stimulate the immune system to recognize GM2, GD2 and GD3 that are present primarily on sarcoma cells	II	Completed
NCT00001566	Dendritic cells + indinavir sulfate	Pediatric ES Rhabdomyosarcoma	Peptide-pulsed antigen presenting cell (APC) vaccine is injected into patients. Following chemotherapy, infusion of harvested T cells followed by infusion of peptide-pulsed APC vaccinations. IL-2 is administered on the same day as T cell /peptide-pulsed infusions	II	Completed
NCT01341496	Tumor cell vaccines + ISCOMATRIX	Sarcoma, melanoma, epithelial Malignancies	The autologous tumor cell vaccine is administered with ISCOMATRIX adjuvant in combination with metronomic oral cyclophosphamide and celecoxib in patients undergoing thoracic metastasectomy	I	Terminated
NCT03357315	Mix vaccine	Metastatic sarcomas	Patients received mix vaccine (experimental group) or not received treatments (control group). The aim of the study is to evaluate safety and efficacy of mix vaccine on small metastases of sarcoma	I/II	Completed

The main over-expressed CTAs in sarcomas are: NY-ESO-1, MAGE, PRAME, BAGE, CAGE; all of them may be excellent candidates for vaccines and for genetically modified adoptive T cells therapy as previously stated [58, 97–110]. One of the most immunogenic CTAs, is NY-ESO-1. It is over-expressed in many types of tumors, in sarcomas it is mainly expressed in synovial sarcomas (85%), myxoid/round cells and liposarcoma [101–104]. Raza et al. showed that the administration of NY-ESO-1 vaccine alone is not enough effective because of negative effect of the suppressive tumor microenvironment. Thus, the combination with an immunologic adjuvant is warranted [102]. A placebo-controlled clinical trial evaluated the safety and immunogenicity of recombinant NY-ESO-1 vaccine with ISCOMATRIX, a saponin-based adjuvant that induces a strong T-cell-based immune response. The vaccine was tested on patients with sarcoma and melanoma. The vaccine was well tolerated and active giving a serological response in all patients and 10/16 patients had a delayed-type hypersensitivity (DTH) response [105]. Another phase I clinical trial (NCT00027911), whose results have not been published yet, used NY-ESO-1 peptide vaccine and sargramostim (GM-CSF) in patients with advanced soft tissue sarcoma expressing NY-ESO-1. Sargramostatin, a colony-stimulating factor, increases the number of immune cells found in bone marrow or peripheral blood and it increases the efficacy of vaccine.

MAGE, Melanoma-associated antigen 3 (MAGE-A3), was the first human tumor-associated antigen to be identified, expressed in the placenta, germline cells and over expressed in various tumors including sarcomas (in particular osteosarcoma and synovial sarcoma). MAGE is a potential target for vaccines and T cells therapy like NY-ESO-1 [106–108]. A pre-clinical study investigated the expression of MAGE-A3 antigen, in several sarcoma cell lines; MAGE-A3 is found highly expressed also in UPS and MF and high expression of the MAGE-A3 protein correlates with worse overall survival [109].

The over-expression of CTAs in sarcomas appears related to epigenetic mechanisms such as hypomethylation of the gene promoters [101–104], for this reason some clinical trials are exploring the efficacy of epigenetic compounds as decitabine (5-aza-2-deoxycytidine) in combination with cancer vaccines (eg NCT01241162) [103]. Epigenetic mechanisms are at the basis of the up regulation of CTAs. Some pre-clinical studies observed that the use of epigenetic compounds such as decitabine, up-regulates NY-ESO-1, LAGE-1, SSX, and MAGE -A10 in sarcoma cell lines [103, 104]. In fact, an interesting phase I clinical trial (NCT01241162) uses decitabine followed by a DCs vaccine. The DCs pulsed with peptides mixes, derived from NY-ESO-1, MAGE-A1, and MAGE-A3, are injected in young patients with synovial sarcoma, osteosarcoma, rhabdomyosarcoma, ES and neuroblastoma. In this study, a CD4+ but not a CD8+ response was registered. In addition, patients with sarcoma had not a long-term control of the disease compared to patients with neuroblastoma who had better results [111].

Another strategy for designing vaccines is to use specific peptides derived from fusion proteins. For example, synovial sarcoma is characterized by chromosomal translocation (X, 18; p11, q11), that triggers SYT-SSX or SS18-SSX fusion protein [96, 112–114]. In a clinical study, a vaccine with SYT-SSX fusion peptide fragment was administered in 21 patients with synovial sarcoma. The combination with interferon alpha ameliorated the disease control rate increasing the number of stable disease (6 out of 12 patients treated) compared to the vaccine-single agent arm (1 out of 9 patients had ST). Other studies show that addition of adjuvants to vaccine administration, as IL-2 and GM-CSF, heat shock proteins and radiation can improve the immune response against cancer in the host and increase the overall survival [17, 115].

**Fig. 2:**
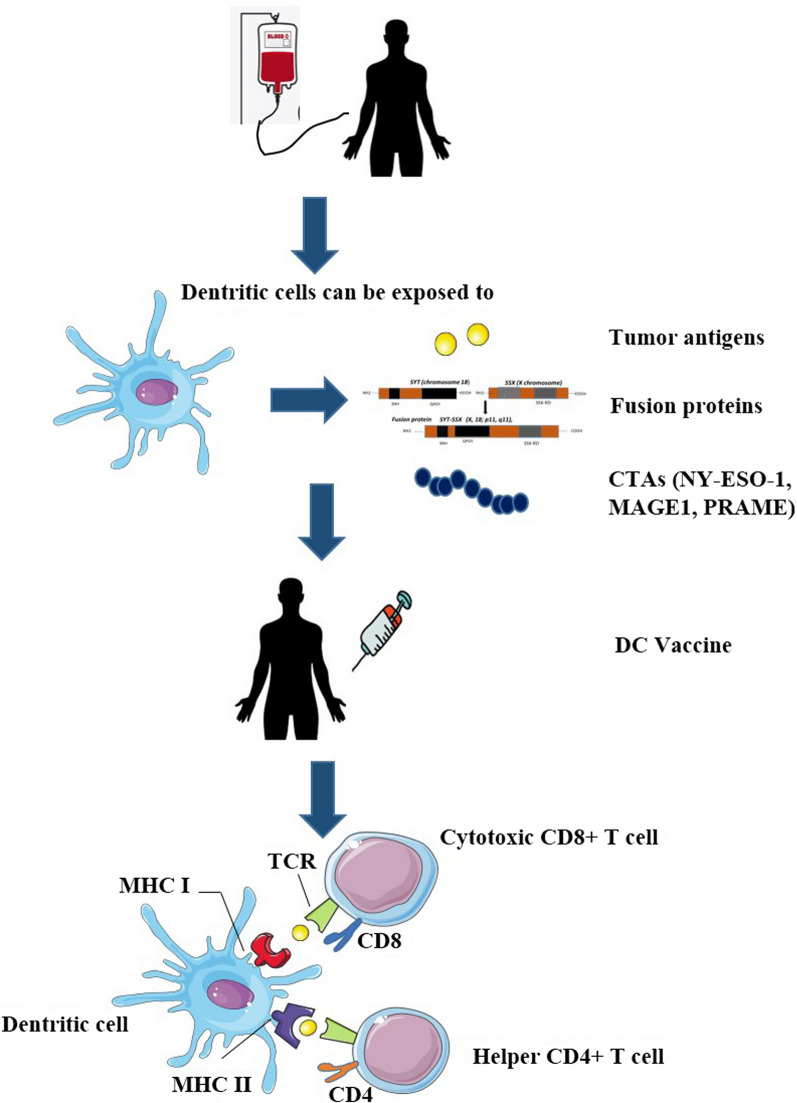
An immunological strategy for designing a vaccine is to use dendritic cells to trigger the immune response process towards cancer cells. Dendritic cells from peripheral blood are extracted through leukapheresis, then stimulated with tumor specific antigens, fusion proteins or pulsed with CTAs antigens or peptides mixes, derived from NY-ESO-1, MAGE-A1, PRAME, and finally reintroduced into the patient. DCs present the antigens through MHC class I and II to lymphocytes. Tumor antigens can be recognized by both CD8+ and CD4+ T lymphocytes, in the presence of costimulatory molecules necessary for their activation. Once T CD8+ lymphocytes have differentiated into effector CTLs, they can kill tumor cells even in the absence of co-stimulation or contribution from helper T cells

Another strategy to build a vaccine is through the use of autologous APCs, e.g. DC-based vaccines (Fig. 2). Patients’ autologous APC, as DCs, can be actively extracted through leukapheresis, then stimulated with tumor specific antigens and finally reintroduced into the patient. They present the antigens through MHC class I and II to the CD4+ and CD8+ lymphocytes triggering the immune response process towards cancer cells [116, 117]. This process is called cross-presentation or cross-priming, since a cell type (i.e. the DCs) presents antigens from another cell (i.e. the tumor cell), activating specific T lymphocytes. Once T CD8+ lymphocytes differentiate into effector CTLs, they can kill tumor cells even in the absence of co-stimulation or contribution from helper T cells. This approach circumvents immunoevasion based on lack of immunologic stimulation due to the absence of tumor antigen presentation. In fact, the downregulation of MHC is one of several complexes mechanisms of cancer immune system evasion [118–121]. MHC expression has also been analyzed in sarcomas and it is low in liposarcomas and synovial sarcomas [117–119]. Other studies showed that down-regulation of MHC class I in STSs, BSs and ESs correlates with a worse prognosis [121–123].

In a phase I clinical trial, STS patients are injected with DC vaccines in combination with radiation. Radiation aims at increasing the release of antigens to DCs inside the tumor site. In this study, it was observed that the combined treatment causes an accumulation of CD4+ T cells in the tumor compared to the administration of the vaccine only. The accumulation of CD4+ T cells positively correlates with tumor-specific immune response [112]. In another study, 16 patients with rhabdosarcoma and ES were treated with a DC vaccine. The DCs collected from the patients, were exposed to fusion proteins, specific for rhabdosarcoma and ES and administered with IL-2, as adjuvant. However, the results obtained were very limited [124]. Another phase I clinical study, administered engineered DCs, modified through LV305, a lentiviral vector inducing the expression of the NY-ESO-1 antigen [125]. The study was conducted on 39 patients of which 24 with sarcoma; the remaining patients had melanoma (6), ovarian (8) and lung cancer (1). One patient with synovial sarcoma had a partial response lasting 36 months, 14 patients had SD. Median PFS was 2.8 months in patients with synovial sarcomas and 4.6 months in patients with other sarcoma histotypes. There were no grade 3 or 4 adverse events, indicating a good tolerance. In 57% of sarcoma patients an anti-NY-ESO-1 response was detected, towards CD4 + and/or CD8 + T cells. In an exploratory analysis, the anti-NY-ESO-1 immune response correlated with improved 1-year survival.

Finally, ganglioside vaccines have been explored in sarcoma patients. GD2, tumor-specific protein, is suitable to immunotherapy through monoclonal antibodies or with artificial T cell receptors [126, 127]. Vaccines anti GD2 are tested in melanoma and sarcoma patients. A phase 2 clinical trial used a trivalent ganglioside vaccine on 136 patients with metastatic sarcoma. Median PFS was 6.4 months, but no significant outcome differences were observed between vaccine and placebo-treated patients [127].

## Biomarkers in sarcomas

Various clinical trials suggest that immunological therapies in sarcomas could be an interesting future treatment option for some histotypes. In this context, the selection of patients who can respond to immunotherapy is a crucial issue. However, to date, the major data on sarcomas concern biomarkers predictive of response to chemotherapy [128–133]. In particular, some recently discovered biomarkers as TOP2A and TLE3 have been identified as potential predictors of response to anthracyclines and taxanes. MGMT, RMM1, TUBB3 have been associated with response to regimens containing alkylating agents [131], gemcitabine or taxanes, respectively [132]. Of note, our research group is currently conducting an observational study to investigate whether MGMT expression levels or MGMT promoter methylation may represent a predictive marker for dacarbazine sensitivity in leiomyosarcoma and solitary fibrous tumours. Very little is known about biomarkers for cancer immunotherapy in sarcoma. To date, the main proposed biomarkers of response to immunotherapy are: high expression of PD-L1, high concentration of TILs in tumour samples, high tumor mutation burden (TMB) and microsatellite instability (MSI).

### PD-L1 / PD-1

The PD-L1 and PD-1 expression in the different subtypes of sarcoma and their possible correlation with the immune checkpoints inhibitors has already been treated in this review [14–25]. However, we emphasize that the discordant and not reproducible data regarding PD-L1 expression could be related to the small size of analyzed series and/or the use of different antibodies [134–136].To date, PD-L1 cannot be considered as an effective predictive biomarker to select patients with sarcoma for treatment with ICIs [135]. Furthermore, the techniques to evaluate the PD-L1 expression in sarcoma patients should be improved and standardized [136].

### Tumor infiltrating lymphocytes (TILs)

The role of TILs has been already discussed in a previous section. We strongly believe that efforts should be made to better understand the role of TILs (including T and NK cells) as 1. potential predictive biomarker of response to immunotherapy, as well as 2. modulating factors to increase response to treatment. Furthermore, the correlation between PD-L1 expression and TILs in STSs should be evaluated. In fact, although a high number of CD8+ T cells do not seem to be a clear prognostic marker of survival in patients with STSs, high PD-L1 expression on TILs has been related to worse survival rates in these patients, suggesting that PD-1/PD-L1 pathway in STSs might prompt cancer progression through T-cell inhibition [36–42].

### Mutation burden

Tumor mutation burden (TMB) is a well-recognized predictive factor of response to immunotherapy in different neoplasms including melanoma, lung. Snyder et al*.* [137] have shown that the treatment with ipilimumab, (antibody anti-CTLA-4) was significantly more effective in patients with melanoma carrying more than 100 mutations per coding genome than those with a lower mutation rate. The CheckMate 227 study reported that patients affected by advanced NSCL and elevated TMB (of at least 10 mutations per megabase), treated with first line therapy nivolumab/ipilimumab showed a significant longer PFS than the counterpart with a lower TMB, suggesting the role of TMB as a biomarker for patient selection [138]. Some studies have shown a low or intermediate mutation burden in osteosarcoma and in epithelioid sarcomas compared to other cancers [139]. In particular, it has been reported that the epithelioid sarcoma has a mutation rate in coding regions similar to ovarian cancer; this could be exploited for treatment with immune checkpoint inhibitors [140, 141]. Treatment with ICIs can probably be more effective in patients with hypermutated sarcoma, but larger studies are needed to validate the predictive role of TMB in sarcomas.

### dMMR/MSI

Mismatch repair deficiency (dMMR) and microtellite instability (MSI) are interesting biomarkers used in several solid tumors to predict response to immunotherapy [142–147]. MMRs are DNA mismatch repair enzymes. When one or more of these enzymes is not expressed or dysfunctional, a mismatch repair deficiency can occur. The MMR complex deficiency can also determine the instability of microsatellites (stretches of short sequences of approximately 16 nucleotide repeated and distributed throughout the genome). Tumors with dMMR or MSI have been reported to be sensitive to PD-1/PD-L1 inhibitors, particularly pembrolizumab [17, 18]. In particular, this correlation has been reported in colorectal cancer (CRC). It is known that the CRC with high MSI manifests an inflammatory phenotype that generates an endogenous immune response, which is counteracted by the expression of inhibitory immune signals such as PD1/PDL1. Based on these considerations, CRCs with high MSI seem to be particularly sensitive to immunotherapy. MSI/dMMR may be predictive biomarkers also in sarcomas, where to date there are very few and conflicting data. Large and prospective trials are needed to address the role of dMMR/MSI in sarcomas.

## Immunological features of sarcomas

Over the past 10 years, attempts to use immunotherapies in the treatment of cancer has exploded. The main theories behind the concept of immune therapy are based on two fundamental concepts: immune-surveillance and immune-editing [148, 149]. Immuno-surveillance is a process of the immune system whereby abnormal cells are recognized and destroyed to prevent cancer formation in the body. Studies have shown that patients with either impaired or suppressed immune system are more prone to develop cancer. For example, the Kaposi’s sarcoma caused by human herpesvirus 8 is extremely rare in the general population, but its incidence is significantly increased in individuals with immune-deficiencies [150].

Immuno-surveillance primarily functions as a component of a more general process of cancer immune-editing.

Cancer immune-editing consists of 3 phases:

- Elimination;
- Equilibrium;
- Escape.

**Fig. 3:**
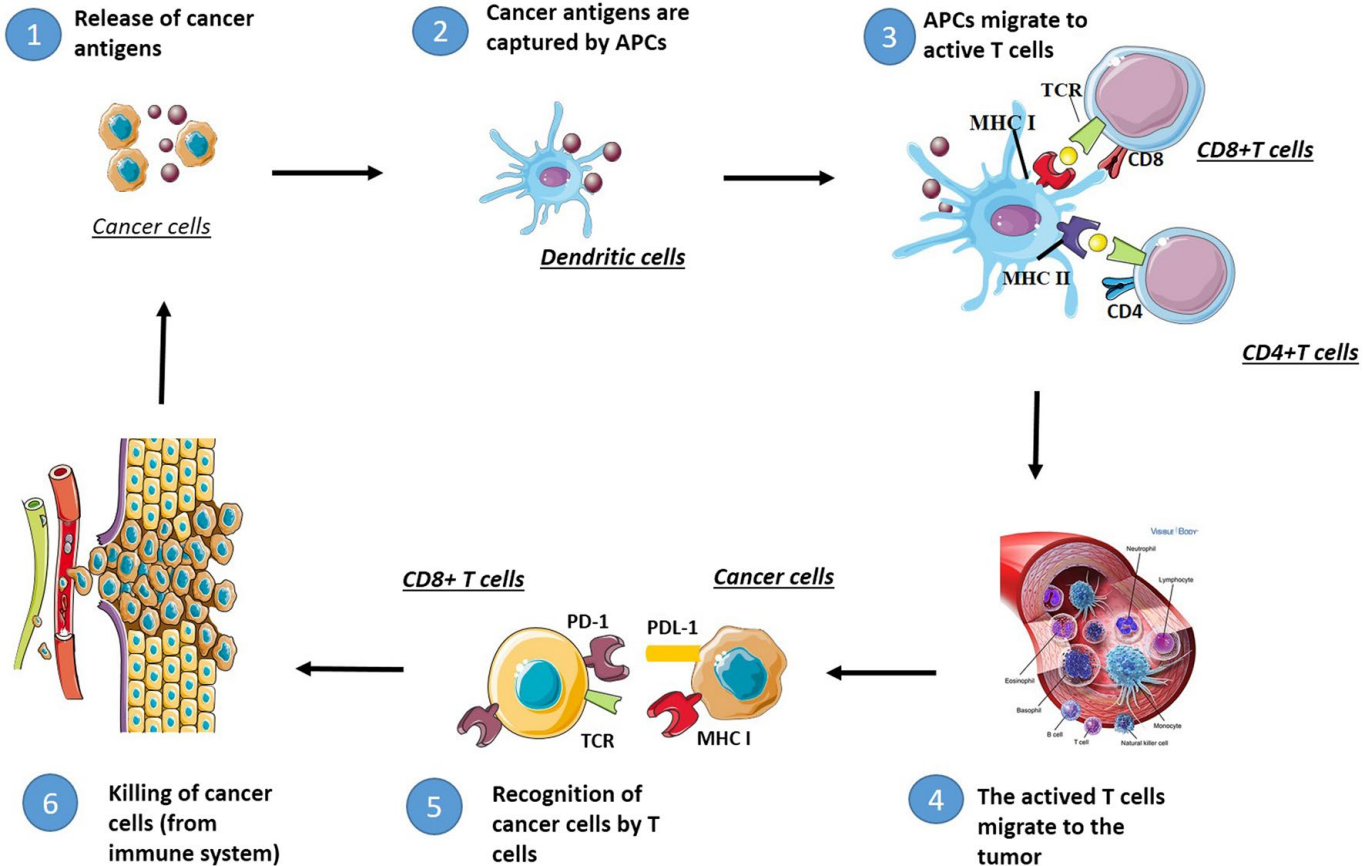
Role of immune system in host/cancer interactions–

In the elimination phase, the immune system recognizes and eliminates cancer cells. In this phase, tumor cells release highly immunogenic antigens that are captured and processed by APCs like macrophages and DCs. APC cells migrate in order to activate T cells (adaptive immunity) by presenting the tumor antigens on MHC molecules. The activated T cells migrate toward the tumor where they proliferate and release pro-inflammatory cytokines leading to tumor cells death via classical pathways. The role of TILs, the possible correlation with the overall survival, and their potential role as prognostic marker has been previously reported in this review [36–42] (Fig. 3).

In the context of sarcomas, the role of the immune system and the potential interactions with the tumor microenvironment have been investigated in ES by Berghuis et al*.* Forty different expression profiles of chemokines in therapy-naive ES patients have been analyzed. They observed that the main tumor infiltrating T cells were CD8 + lymphocytes and that they positively correlated with different pro-inflammatory chemokines expression (CXCR3- and CCR5-ligands CXCL9, CXCL10 and CCL5). These findings suggested that an inflammatory immune microenvironment, with high expression of these chemokines, may be very important for T cell recruitment preventing the progression of ES [151]. The elimination phase is followed by an “equilibrium” phase, where the number of proliferating tumor cells equals the number of dying cells because of the action of immune system. In this phase the tumor does not grow but it is still present and remains sub clinical in most cases, because the immune system is able to control either prevent further growth of cancer cells [152]. On the other hand, in the “escape” phase the tumor can overwhelm the immune system going ahead with its growth and clinical manifestation [153–155]. Cancer cells acquire the ability to suppress or evade the immune response, the immune system cannot eliminate and control the growth of tumor, that continue proliferating and spreading. This switch from “equilibrium” to “escape” phase can be due to different mechanisms, including loss/gain of function mutations, epigenetic alterations, affecting genes encoding for pro/anti-apoptotic proteins, MHC, antigen-presenting machinery, etc. This protects tumor cells from death even if an apoptotic stimulus is received. Inactivation of BCL2 pro-apoptotic members family (through mutation) is a known example of this phenomenon in sarcomas. Another frequent tumor escape mechanism is the MHC I loss. In this case tumor cells do not express MHC I and therefore antigens are no longer being presented to CD8+ lymphocytes. This phenomenon has been extensively described in sarcomas [121]. A key role in the immuno-evasion process is also played by the immune-checkpoints inhibitors, such as PD-1 and PDL-1, which are usually overexpressed by tumor cells thus effectively blocking T cells activity. In order to evade the immune system, tumor cells express high levels of inhibitory checkpoint molecules as PDL-1 or CD80 consequently stopping the immune system response. To date, specific antibodies have been developed to target these proteins as already discussed in this work.

## Conclusions

The goal of immunotherapy treatment is to restore the immune system ability to recognize cancer cells and eliminate them effectively, overcoming the mechanisms by which tumors suppress the immune response. Sarcomas are rare tumors, ubiquitous and heterogeneous with behaviors that differ mainly in relation to the anatomic site of origin. In localized disease, the overall survival, the disease free survival and the quality of life of patients are strongly influenced by the adequacy of the surgical approach and the overall therapeutic strategy. In locally advanced and metastatic setting the outcomes are still poor, despite the several chemotherapy treatments available to date.

Pembrolizumab and nivolumab have been approved by FDA for the treatment of melanoma, non-small cell lung cancer, lymphoma, and urothelial carcinoma. Consequently, the efficacy of these immune-therapeutic drugs has been tested in sarcomas treatment in recent years. However, the study of the immunotherapy approach as well as the identification of biomarkers predictive of response in sarcomas are difficult because of the rarity and heterogeneity of the disease. There are few clinical trials in progress and still many years are needed for their outcome analysis due to the low number of patients enrolled. Cancer vaccines in sarcoma therapy have induced some responses; future studies should focus in the identification of more specific tumor antigens, to limit the toxicity of vaccines and identify optimal treatment strategies. Promising results have been achieved with the CAR-T therapies but they should be confirmed in larger cohorts.

In conclusion, the main goal for the future clinical trials on immunotherapy in sarcomas setting should be to select innovative and specific biomarkers (tumor antigen expression, gene mutations, structural rearrangements, etc.), and to improve multi-institutional collaborations in order to increase patients’ enrollment and increase the quality and the reliability of clinical trials.

## Data Availability

Not applicable.

## Abbreviations

NSCLC: Non-small cell lung cancer
CTAs: Tumor specific antigens
CAR-T: Chimeric antigen receptor T-cells
PD-1: Programmed cell death-1
CD19: Cluster of differentiation 19
CTLA- 4: Cytotoxic T-lymphocyte associated protein-4
ACT: Adoptive T cell transfer
ICIs: Immune checkpoint inhibitors
PD-L1: Programmed death-ligand 1
TMB: Tumor mutation burden
MSI: Micro satellite instability
ORR: Objective response rate
UPS: Undifferentiated pleomorphic sarcoma
PFS: Progression-free survival
CR: Complete response
PR: Partial responses
DDLPS: Dedifferentiated liposarcoma
SS: Synovial sarcoma
LMS: Leiomyosarcoma
ES: Ewing Sarcoma
DC: Dedifferentiated chondrosarcoma
EPS: Epithelioid sarcoma
FD: Food and drug administration
NY-ESO 1: New York esophageal squamous cell carcinoma 1
STS: Soft-tissue sarcomas
SD: Stable disease
ASCO: American Society of Clinical Oncology
PFS: Progression-free survival
ASPS: Alveolar soft part sarcoma
GIST: Gastrointestinal stromal tumor
DDLPS: De-differentiated liposarcoma
TILs: Tumor-infiltrating lymphocytes
TCR T: T cell receptor
DNA: Deoxyribo nucleic acid
Ig4: Immunoglobulin 4
MHC: Major histocompatibility complex
IL: Interleukin
HER2: Human epidermal growth factor receptor 2
GD2: Disialoganglioside
IGF-1R: Insulin-like growth factor 1
AP1903: 2,2'-[1,2-Ethanediylbis[imino(2-oxo-2,1-ethanediyl) oxy-3,1-phenylene[(1R)-3-(3,4-dimethoxyphenyl)propylidene]]] bis[(2S)-1-[(2S)-1-oxo-2-(3,4,5-trimethoxyphenyl)butyl]-2-piperidinecarboxylate
AACR: Association for Cancer Research
DC: Dendritic cells
PAP: PROSTATE
BCG: Bacillus Calmette-Guérin
HSV-1: Herpes simplex virus type 1
GM-CSF: Granulocyte–macrophage colony-stimulating factor
MAGE: Melanoma-associated antigen
PRAME: Nuclear receptor transcriptional regulator
BAGE: B melanoma antigen
CAGE: Cancer-associated antigen gene
DTH: Delayed-type hypersensitivity
APC: Antigen presenting cells
CTLs: Cytotoxic T lymphocytes
HLA: Human leukocyte antigens
TMB: Tumor mutational burden
MSI: Microsatellite instability
MDSC: Myeloid-derived suppressor cells
SC: Subcutaneously
MK3475: Pembrolizumab
T-VEC: Talimogene laherparepvec
LY3012207: Olaratumab
PBMCs: Peripheral blood mononuclear cells
PSMA: Prostate-specific membrane antigen
MTD: Maximum tolerated dose
DLT: Dose limiting toxicities
PAP: Prostatic Acid Phosphatase
